# Supplementary material for: Efficiency of Digital Photolithographic Synthesis of Large, High-Quality DNA Libraries and Microarrays using a Guanine O6 Dephosphitylation Strategy
Source: Commun Chem. 2025 Oct 31;8:321. doi: 10.1038/s42004-025-01755-0 (PMC12578808; doi:10.1038/s42004-025-01755-0)
Supplement: Supplementary file 2 — Supplementary material [file 42004_2025_1755_MOESM2_ESM.pdf]

Supplementary Material for

Efficiency of Digital Photolithographic Synthesis of Large, High-Quality DNA Libraries and Microarrays using a Guanine O[6] Dephosphitylation Strategy

Santra Santhosh[1,2], Sharon Istvánffy[2,3], Omer Sabary[4], Eitan Yaakobi[4], Maya Giridhar[2], Jürgen Behr[2], Mark M. Somoza[2,5,6] *

[1] Technical University of Munich, TUM School of Natural Sciences, Garching, Germany

[2] Leibniz Institute for Food Systems Biology at the Technical University of Munich, Lise-Meitner-Straße 30, 85354 Freising, Germany

[3] Technical University of Munich, TUM School of Life Sciences, Freising, Germany

[4] The Henry and Marilyn Taub Faculty of Computer Science, Technion, 3200003, Haifa, Israel

[5] Chair of Food Chemistry and Molecular Sensory Science, School of Life Sciences, Technical University of Munich, Lise-Meitner-Straße 34, 85354 Freising, Germany

[6] Department of Inorganic Chemistry, Faculty of Chemistry, University of Vienna, Josef-Holaubek-Platz 2, 1090 Vienna, Austria

* To whom correspondence should be addressed; email: mark.somoza@univie.ac.at

Supplementary Tables

Supplementary Table 1. Sequences used for gel analysis and hybridization analysis

**Sequence 5'→3'**	**Name**
GTCATCATCATGAACCACCCTGGTCT	26mer
GTTAAGCGAAGAAGAAAGTAGCGTGGCGCACAGTTGCCCAATCAATTACACCCTCATTTCT	Mixed base 61mer – with G
TTTAATCTAATAATAAATTATCTTTTCTCACATTTTCCCAATCAATTACACCCTCATTTCT	Mixed base 61mer – T instead of G
TTTTTTTTTTTTTTTTTTTTTTTTTTTTTTTTTTTTTTTTGTTTTTTTTTTTTTTTTTTT	G_{20}-T_{60}
TTTTTTTTTTTTTTTTTTTTTTTTTTTTTTTTTTTTTTTGGTTTTTTTTTTTTTTTTTTT	$G_{20,21}$-T_{60}
TTTTTTTTTTTTTTTTTTTTTTTTTTTTTTTTTTTTTTTAATTTTTTTTTTTTTTTTTTT	$A_{20,21}$-T_{60}
GACCAGGGTGGTTCATGATGATGAC	QC25
GAAATGAGGGTGTAATTGATTGGGCAACTGTGCGCCACGCTACTTTCTTCTTCGCTTAAC	ECOBioD2_60mer
GATTTAGGTTTACAAGTCTACACCGAATTAACAACAAAAAACACGTTTTGGAG	ECOBioA1t_53mer

Supplementary Table 2. Synthesis protocol for the cycle of 5'BzNPPOC-dT. The "No Debranching/Normal" protocol is the old protocol, and the "15s Debranching" protocol adds the capping reagents for 15s after the coupling reaction. In the case of other debranching times, only the 15s parameter is changed.

**No Debranching/Normal**					**15s Debranching**				
**Function**	**Mode**	**Pulse**	**Sec**	**Description**	**Function**	**Mode**	**Pulse**	**Sec**	**Description**
**$Coupling**					**$Coupling**				
1/*Wsh	*/PULSE	20	0	"Flush system with anhydrous ACN"	1/*Wsh	*/PULSE	20	0	"Flush system with anhydrous ACN"
2/*Act	*/PULSE	6	0	"Activator"	2/*Act	*/PULSE	6	0	"Activator"
21/*T+Act	*/PULSE	6	0	"T+Activator"	21/*T+Act	*/PULSE	6	0	"T+Activator"
2/*Act	*/PULSE	6	0	"Push to cell with Activator"	2/*Act	*/PULSE	6	0	"Push to cell with Activator"
1/*Wsh	*/PULSE	10	0	"Push to cell with anhydrous ACN"	1/*Wsh	*/PULSE	10	0	"Push to cell with anhydrous ACN"
1/*Wsh	*/PULSE	3	15	"Couple Monomer"	1/*Wsh	*/PULSE	3	15	"Couple Monomer"
1/*Wsh	*/PULSE	10	0	"Push to cell with anhydrous ACN"	1/*Wsh	*/PULSE	10	0	"Push to cell with anhydrous ACN"

Table continues on the next page.

**$Capping**					**$Capping**				
					13/*Caps	*/PULSE	7	0	“Fill line with debranching reagents”
					12/*WshA	*/PULSE	16	0	“Push debranching reagents to cell with anhydrous ACN”
					12/*WshA	*/PULSE	3	15	“Capping/Debranching”
					12/*WshA	*/PULSE	10	0	“Wash cell with anhydrous ACN”
40/*GasA	*/PULSE	1	20	“Dry Cell”	40/*GasA	*/PULSE	1	20	“Dry Cell”
**$Oxidizing**					**$Oxidizing**				
15/*Ox	*/PULSE	10	0	“Fill line with Oxidizer”	15/*Ox	*/PULSE	10	0	“Fill line with Oxidizer”
12/*WshA	*/PULSE	16	0	“Push Ox to cell with anhydrous ACN”	12/*WshA	*/PULSE	16	0	“Push Ox to cell with anhydrous ACN”
12/*WshA	*/PULSE	3	15	“Oxidize”	12/*WshA	*/PULSE	3	15	“Oxidize”
12/*WshA	*/PULSE	10	0	“Wash cell with anhydrous ACN”	12/*WshA	*/PULSE	10	0	“Wash cell with anhydrous ACN”
17/*AUX	*/PULSE	10	0	“Fill line with exposure solvent”	17/*AUX	*/PULSE	10	0	“Fill line with exposure solvent”
12/*WshA	*/PULSE	20	0	“Push exposure solvent to cell with anhydrous ACN”	12/*WshA	*/PULSE	20	0	“Push exposure solvent to cell with anhydrous ACN”
130/*Event 2 Out	*/PULSE	4	1	“Event 2 Out”	130/*Event 2 Out	*/PULSE	4	1	“Event 2 Out”
12/*WshA	*/PULSE	8	30	“Push with anhydrous ACN”	12/*WshA	*/PULSE	8	30	“Push with anhydrous ACN”
130/*Event 2 Out	*/PULSE	4	1	“Event 2 Out”	130/*Event 2 Out	*/PULSE	4	1	“Event 2 Out”
12/*WshA	*/PULSE	15	0	“Flush System with anhydrous ACN”	12/*WshA	*/PULSE	15	0	“Flush System with anhydrous ACN”

Supplementary Table 3. Number of cycles required for the synthesis.

Synthesis name	Number of unique sequences	Number of synthesis cycles (including T_5 spacer and cleavable dT)
26mer	1	32
Mixed-base 61mer	1	67
G_{20}-T_{60}	1	66
$G_{20,21}$-T_{60}	1	66
$A_{20,21}$-T_{60}	1	66
Mixed-base 61mers complex array	82902	174
qPCR library	8	151
G-rich library for hybridization assay	57	116
Gene Expression	~129000	160

Supplementary Table 4. 40mer homopyrimidine test probes to check the effect of guanosine-modified depurination-based fragmentation on hybridization assay. All the sequences have a T_5-linker at their 3′ end and at the 5′ end a T_5-linker followed by a 25mer reporter sequence (5′-GTCATCATCATGAACCACCCTGGTC-3′). The P0 sequence is the control sequence. P2, P3, and P4 represent "GG", "GGG", and "GGGG", i.e., 2, 3, and 4 sites for O^6-phosphitylation.

**Test probes 5′→3′**	**Name**
TCTCTCTCTCTCTCTCTCTCTCTCTCTCTCTCTCTCTCTC	P0
TCTCTCTCTCTCTCTCTCTCTCTCTCTCTCTCTCTCT**GG**C	P2_02
TCTCTCTCTCTCTCTCTCTCTCTCTCTCTCTCTCT**GG**CTC	P2_04
TCTCTCTCTCTCTCTCTCTCTCTCTCTCTCTCT**GG**CTCTC	P2_06
TCTCTCTCTCTCTCTCTCTCTCTCTCTCTCT**GG**CTCTCTC	P2_08
TCTCTCTCTCTCTCTCTCTCTCTCTCTCT**GG**CTCTCTCTC	P2_10
TCTCTCTCTCTCTCTCTCTCTCTCTCT**GG**CTCTCTCTCTC	P2_12
TCTCTCTCTCTCTCTCTCTCTCTCT**GG**CTCTCTCTCTCTC	P2_14
TCTCTCTCTCTCTCTCTCTCTCT**GG**CTCTCTCTCTCTCTC	P2_16
TCTCTCTCTCTCTCTCTCTCT**GG**CTCTCTCTCTCTCTCTC	P2_18
TCTCTCTCTCTCTCTCTCT**GG**CTCTCTCTCTCTCTCTCTC	P2_20
TCTCTCTCTCTCTCTCT**GG**CTCTCTCTCTCTCTCTCTCTC	P2_22
TCTCTCTCTCTCTCT**GG**CTCTCTCTCTCTCTCTCTCTCTC	P2_24
TCTCTCTCTCTCT**GG**CTCTCTCTCTCTCTCTCTCTCTCTC	P2_26
TCTCTCTCTCT**GG**CTCTCTCTCTCTCTCTCTCTCTCTCTC	P2_28
TCTCTCTCT**GG**CTCTCTCTCTCTCTCTCTCTCTCTCTCTC	P2_30
TCTCTCT**GG**CTCTCTCTCTCTCTCTCTCTCTCTCTCTCTC	P2_32
TCTCT**GG**CTCTCTCTCTCTCTCTCTCTCTCTCTCTCTCTC	P2_34
TCT**GG**CTCTCTCTCTCTCTCTCTCTCTCTCTCTCTCTCTC	P2_36
T**GG**CTCTCTCTCTCTCTCTCTCTCTCTCTCTCTCTCTCTC	P2_38
TCTCTCTCTCTCTCTCTCTCTCTCTCTCTCTCTCT**GGG**CT	P3_03
TCTCTCTCTCTCTCTCTCTCTCTCTCTCTCTCT**GGG**CTCT	P3_05
TCTCTCTCTCTCTCTCTCTCTCTCTCTCTCT**GGG**CTCTCT	P3_07
TCTCTCTCTCTCTCTCTCTCTCTCTCTCT**GGG**CTCTCTCT	P3_09
TCTCTCTCTCTCTCTCTCTCTCTCTCT**GGG**CTCTCTCTCT	P3_11
TCTCTCTCTCTCTCTCTCTCTCTCT**GGG**CTCTCTCTCTCT	P3_13
TCTCTCTCTCTCTCTCTCTCTCT**GGG**CTCTCTCTCTCTCT	P3_15
TCTCTCTCTCTCTCTCTCTCT**GGG**CTCTCTCTCTCTCTCT	P3_17

TCTCTCTCTCTCTCTCTCT**GGG**CTCTCTCTCTCTCTCTCT	P3_19
TCTCTCTCTCTCTCTCT**GGG**CTCTCTCTCTCTCTCTCTCT	P3_21
TCTCTCTCTCTCTCT**GGG**CTCTCTCTCTCTCTCTCTCTCT	P3_23
TCTCTCTCTCTCT**GGG**CTCTCTCTCTCTCTCTCTCTCTCT	P3_25
TCTCTCTCTCT**GGG**CTCTCTCTCTCTCTCTCTCTCTCTCT	P3_27
TCTCTCTCT**GGG**CTCTCTCTCTCTCTCTCTCTCTCTCTCT	P3_29
TCTCTCT**GGG**CTCTCTCTCTCTCTCTCTCTCTCTCTCTCT	P3_31
TCTCT**GGG**CTCTCTCTCTCTCTCTCTCTCTCTCTCTCTCT	P3_33
TCT**GGG**CTCTCTCTCTCTCTCTCTCTCTCTCTCTCTCTCT	P3_35
T**GGG**CTCTCTCTCTCTCTCTCTCTCTCTCTCTCTCTCTCT	P3_37
TCTCTCTCTCTCTCTCTCTCTCTCTCTCTCTCTCT**GGGG**C	P4_02
TCTCTCTCTCTCTCTCTCTCTCTCTCTCTCTCT**GGGG**CTC	P4_04
TCTCTCTCTCTCTCTCTCTCTCTCTCTCTCT**GGGG**CTCTC	P4_06
TCTCTCTCTCTCTCTCTCTCTCTCTCTCT**GGGG**CTCTCTC	P4_08
TCTCTCTCTCTCTCTCTCTCTCTCTCT**GGGG**CTCTCTCTC	P4_10
TCTCTCTCTCTCTCTCTCTCTCTCT**GGGG**CTCTCTCTCTC	P4_12
TCTCTCTCTCTCTCTCTCTCTCT**GGGG**CTCTCTCTCTCTC	P4_14
TCTCTCTCTCTCTCTCTCTCT**GGGG**CTCTCTCTCTCTCTC	P4_16
TCTCTCTCTCTCTCTCTCT**GGGG**CTCTCTCTCTCTCTCTC	P4_18
TCTCTCTCTCTCTCTCT**GGGG**CTCTCTCTCTCTCTCTCTC	P4_20
TCTCTCTCTCTCTCT**GGGG**CTCTCTCTCTCTCTCTCTCTC	P4_22
TCTCTCTCTCTCT**GGGG**CTCTCTCTCTCTCTCTCTCTCTC	P4_24
TCTCTCTCTCT**GGGG**CTCTCTCTCTCTCTCTCTCTCTCTC	P4_26
TCTCTCTCT**GGGG**CTCTCTCTCTCTCTCTCTCTCTCTCTC	P4_28
TCTCTCT**GGGG**CTCTCTCTCTCTCTCTCTCTCTCTCTCTC	P4_30
TCTCT**GGGG**CTCTCTCTCTCTCTCTCTCTCTCTCTCTCTC	P4_32
TCT**GGGG**CTCTCTCTCTCTCTCTCTCTCTCTCTCTCTCTC	P4_34
T**GGGG**CTCTCTCTCTCTCTCTCTCTCTCTCTCTCTCTCTC	P4_36
T**GGGG**CTCTCTCTCT**GGGG**CTCTCTCTCTCTCTCT**GGGG**C	G_rich

Quantitative verification of amplifiable sequences in the sample via qPCR

The qPCR protocol we follow for sequencing requires taking 5 ng/µl of the DNA sample to achieve the expected number of PCR cycles.[1] An increase in PCR cycles would result in errors due to PCR bias. We diluted "debranching" and "no debranching" to 5 ng/µl. The same volume of water taken to dilute "debranching" to 5 ng was used to dilute "no debranching HC" for comparative analysis. A serial dilution of the samples "debranching" and "no debranching HC" (X:Y, where X = volume of sample, Y = final volume) was taken for qPCR analysis to verify the actual concentration of amplifiable sequences in the sample pool.

Supplementary Table 6. Concentration measured using Nanodrop of the crude sample dissolved in 30 µl after lyophilization. The sequences provided in Supplementary Table 4 are synthesized with and without incorporating the debranching step after coupling.

Sample	Concentration (ng/µl)
Debranching	20.61
No debranching	139.65

Supplementary Table 7. Concentration of the samples measured using Nanodrop and calculated with qPCR using the relative comparison method

Sample	Concentration measured with Nanodrop (ng/µl)	Concentration calculated with qPCR (ng/µl)	Ratio Nanodrop to qPCR
Debranching	4.69	4.8	0.97
Debranching 1:2	2.154	1.87	1.15
Debranching 1:5	0.784	0.79	0.99
Debranching 1:10	0.375	0.53	0.71
No debranching HC	36.435	0.471	77
No debranching HC 1:2	15.076	0.332	45
No debranching HC 1:5	6.076	0.272	22
No debranching HC 1:10	2.806	0.2528	11
No debranching	4.167	0.261	16

Supplementary Table 8s. Calculation table for finding the real concentration with respect to the reference sample, debranching.

Sample	Concentration from Nanodrop (ng/µl)	Mean Cq value	ΔCq (Mean Cq_{sample} – Mean $Cq_{debranching}$)	Log_2ΔCq
Debranching	4.69	8.20	0.00	1
Debranching 1:2	2.154	9.69	1.49	0.36
Debranching 1:5	0.784	11.24	3.04	0.12
Debranching 1:10	0.375	12.18	3.98	0.063

Supplementary Table 5. Cleavable sequences used for qPCR and sequencing analysis to verify the effect of incorporating debranching. The 3′ end had 5′-AGATCGGAAGAGCACACGTCT-3′, and the 5′ end had 5′-ACACGACGCTCTTCCGATCT-3′ for primer binding as adapter 2 and 1, respectively. The sequence design is as follows: 5′-Adapter1->Test_probe->Barcode->Adapter2-3′.

Test_probe (5′→3′)	Barcode (5′→3′)	Name
CTCTCTCTCTCTCTCTCTCTCTCTCTCTCTCTCTCTCTCTCTCTCTCTCTC	ATGTAGATTGC	s0
**GGG**TCTCTCTCTCTCTCTCTCTCTCTCTCTCTCTCTCTCTCTCTCTCTCTC	CCGCCGATAGA	s1
TCTCTCTCTC**GGG**TCTCTCTCTCTCTCTCTCTCTCTCTCTCTCTCTCTCTC	GCAGATGCGTT	s2
TCTCTCTCTCTCTCTCTCTC**GGG**TCTCTCTCTCTCTCTCTCTCTCTCTCTC	GACGTGATCCT	s3
TCTCTCTCTCTCTCTCTCTCTCTCTCTCTC**GGG**TCTCTCTCTCTCTCTCTC	CAGTCTATGTA	s4
TCTCTCTCTCTCTCTCTCTCTCTCTCTCTCTCTCTCTCTC**GGG**TCTCTCTCTC	GTGCATCCGAC	s5
TCTCTCTCTCTCTCTCTCTCTCTCTCTCTCTCTCTCTCTCTCTCTCTCTC**GGG**	ATGACTTCGTA	s6
**GGG**CTCTCTCTCTCTCTCTC**GGG**CTCTCTCTCTCTCTCTCTCTCTCTCTC**GGG**	GCCAGCATAGC	s7

Supplementary Table 9. Overhang primer sequences for adding Illumina indices. Bases in bold indicate the index region for Illumina multiplexing.

Primer	Sequence (5'-3')
2FUF	AATGATACGGCGACCACCGAGATCTACACTCTTTCCCTACACGACGCTCTTCCGATCT
2RIF-GM15	CAAGCAGAAGACGGCATACGAGAT**TGACAT**GTGACTGGAGTTCAGACGTGTGCTCTTCCGATCT
2RIF-GM17	CAAGCAGAAGACGGCATACGAGAT**CTCTAC**GTGACTGGAGTTCAGACGTGTGCTCTTCCGATCT

Supplementary Figures

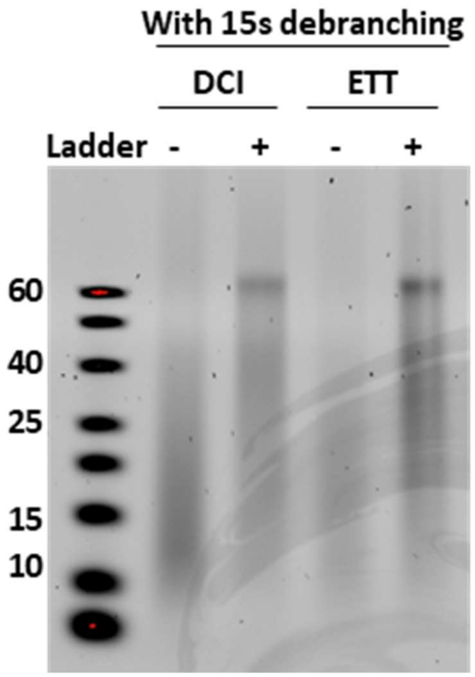

Supplementary Figure 1. Verification of the effect of incorporating 15s debranching step for complex array synthesis. Complex array of ~80,000 unique 61mers synthesized with different coupling activators DCI and ETT, and with and without incorporating 15s debranching step. For all the gel images, same volume of the sample was taken.

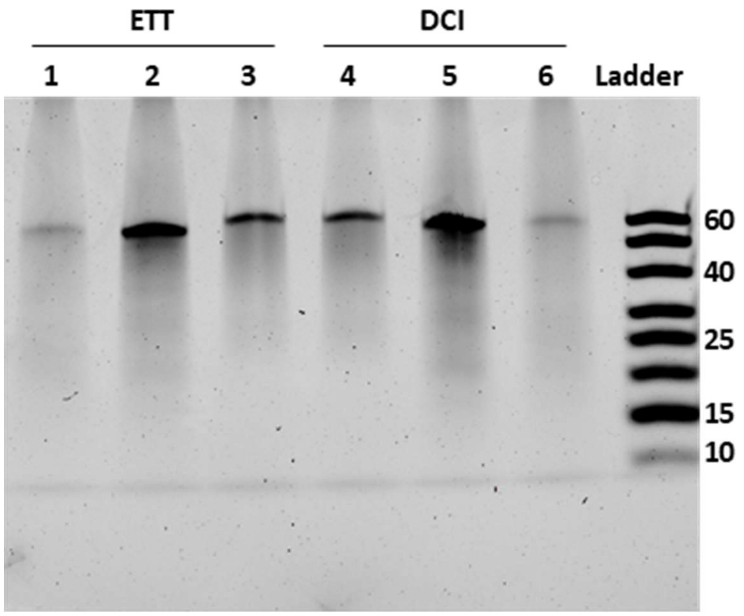

Supplementary Figure 2. Verification of acid-mediated depurination-based fragmentation on microarray. Lane 1&6: Mixed-base 61mer, lane 2&5: mixed-base 61mer – T instead of G, lane 3&4: $A_{20,21}$-T_{60} homopolymer. ETT is more acidic than DCI, which could initiate acid-based depurination during synthesis.

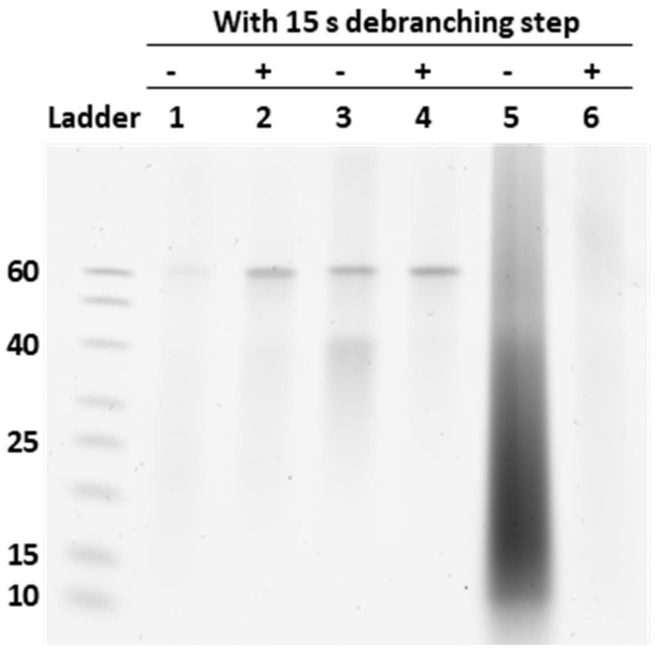

Supplementary Figure 3. Gel image in correct light exposure for verification of the effect of incorporating a 15s debranching step. Lane 1&2: Mixed-base 61mer, lane 3&4: $G_{20,21}$-T_{60} homopolymer and lane 5&6: complex array of ~80,000 unique 61mers. For all the gel images, the same volume of the sample was taken.

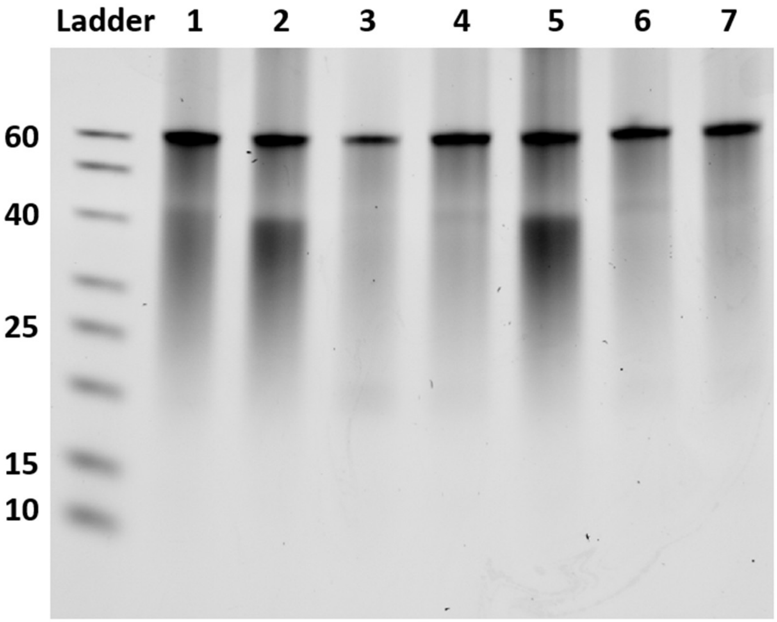

Supplementary Figure 4. Analysis of the extent of O^6-phosphitylation-induced fragmentation in 60mers. Lane 1: Test sequence with only 1 guanosine ($G_{20}T_{60}$) without debranching, lanes 2-7: test sequence with two guanosines ($G_{20,21}$-T_{60}) synthesized under different conditions. Lane 2: normal cycle (without debranching) showing extensive fragmentation than lane 1. Lane 3: with 15s debranching, showing minimal fragmentation. Lane 4: with Unicap coupling instead of debranching in the normal cycle (post-coupling), showing reduced fragmentation, but a residual 40mer band. Lane 5: 4× acetonitrile wash post coupling in normal cycle, no change in fragmentation relative to normal wash. Lanes 6&7: additional synthesis cycles of normal or debranching cycles, respectively, after $G_{20,21}$-T_{60} sequence synthesized with debranching followed and without deprotecting 5′-BzNPPOC (equivalent to 5′ capping), showing a faint 40mer band in the former case and absence of 40mer band in the latter case. All the sequences were synthesized on the microarray following the "no debranching/normal" or "15s debranching" protocols as described in Fig. 1 in the main manuscript and Supplementary Table 2. The same volume of the sample was taken for PAGE analysis. The cleaner gels with UniCap (lane 4) result from terminated branch extension after phosphitylation with UniCap phosphoramidite (lacking a 5′ hydroxyl), producing very short 3′ guanosine depurination products (3′ guanine-UniCap) that are too short to be visualized on the gel. However, UniCap cannot prevent O^6-phosphitylation of guanosine. Similarly, the loss of the dark smears in the additional-cycle reactions (lane 6) reflects termination of branch extension without photodeprotection, yielding 3′-guanine-dT. Fragment distributions differ between $G_{20}T_{60}$ (lane 1) and $G_{20,21}T_{60}$ (lane 2) synthesized with the normal cycle (without debranching). $G_{20}T_{60}$ produces: i) one 20mer from the 3′ array end, ii) one 40mer α,β-unsaturated aldehyde product, iii) one 40mer from the 3′ guanine depurination product for $G_{20}T_{60}$. $G_{20,21}T_{60}$ produces: i) one 20mer from the 3′ array end, ii) one 40mer α,β-unsaturated aldehyde product, iii) two 40mers from the 3′ guanine depurination product for $G_{20,21}T_{60}$, giving a 1:2 ratio and 1:3 of 20mer to 40mer products, respectively, in analogy with the simpler sequence fragmentation products illustrated in Fig. 2 of the main manuscript. Because GelRed binds strongly to T-homopolymers, these appear on the gel at 1:4 vs 1:6 effective intensity ratios, explaining the weak 20mer band and the stronger 40mer band. Although UniCap (lane 4) allows ~80 potential couplings versus 30 in the additional-cycle synthesis (lane 6), both show similar 40mer intensities (lane 4 > lane 6). This likely reflects the reduced O^6-phosphitylation efficiency at the late stage as the guanosines become buried under many terminal thymines and are less accessible to incoming phosphoramidites.

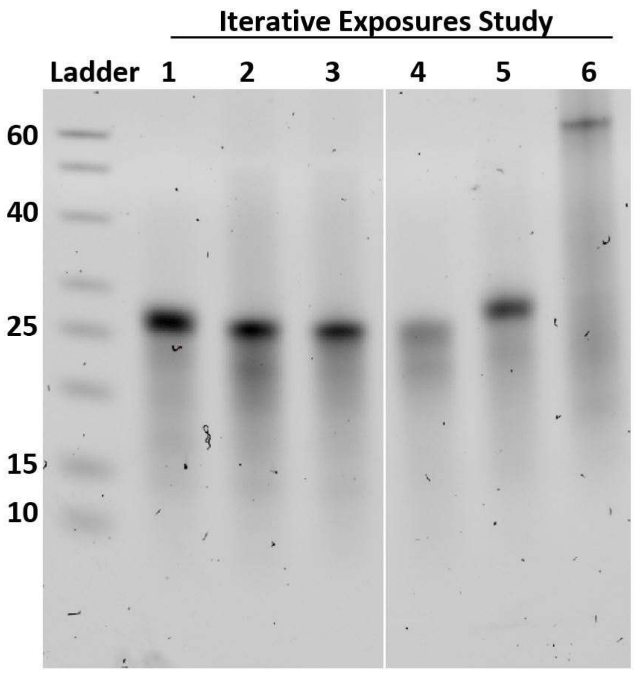

Supplementary Figure 5. Effect of iterative exposures of reagents on 26mer. Lane 1: 26mer; Lane 2: 100 fake exposures of oxidizer; Lane 3: 100 fake exposures of UV and exposure solvent (1% imidazole in DMSO); Lane 4: 100 fake exposures of oxidizer and UV and exposure solvent (1% imidazole in DMSO); Lane 5: 100 fake exposures of oxidizer and coupling activator; Lane 6: Mixed-base 61mer without any additional exposures.

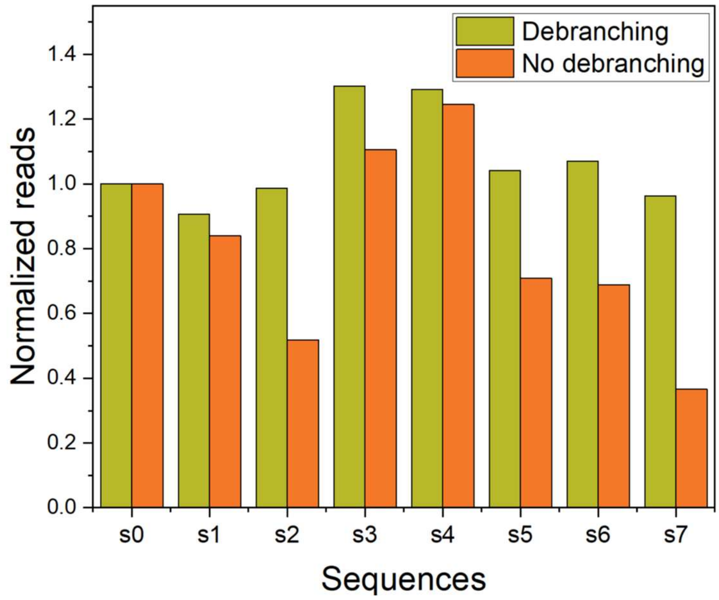

Supplementary Figure 6. Comparison of the effective rate of fragmentation in the case of with and without debranching. The filtered reads are normalized to their own s0 value. The effective rate of fragmentation increases as the G-rich region moves from 5′ to 3′ in the case of synthesis without debranching. The substantial reduction in the number of reads for s2 may be due to the presence of four Gs within the barcode. In contrast, the slight increase in the number of reads for s4 could be due to the presence of only two Gs in its barcode, whereas all the other sequences contain three Gs. Refer to Supplementary Table 4 for sequence design.
